# Association Between the Health Locus of Control and Medication Adherence: An Observational, Cross-Sectional Study in Primary Care

**DOI:** 10.3389/fmed.2021.705202

**Published:** 2021-08-16

**Authors:** Hanna-Maria E. Gerland, Tino Prell

**Affiliations:** ^1^Department of Neurology, Jena University Hospital, Jena, Germany; ^2^Center for Healthy Ageing, Jena University Hospital, Jena, Germany

**Keywords:** adherence, depression, community-dwelling population, locus of control, drugs, compliance

## Abstract

**Background:** Medication non-adherence is an important healthcare issue and a common problem. Many predictors of non-adherence have been found in different settings and cohorts.

**Objective:** Evaluate the impact of the health locus of control (HLC) on unintentional/intentional non-adherence in primary care.

**Methods:** In this observational, cross-sectional study, 188 patients (mean age 63.3 ± 14.9 years) were recruited from three primary care practices in Jena, Germany, over 4 months. The study assessed demographic data, self-reported adherence (German Stendal adherence to medication score, SAMS), HLC, and depression.

**Results:** According to the SAMS total score, 44 (27.5%) were fully adherent, 93 (58.1%) were moderately non-adherent, and 23 (14.4%) were clinically significantly non-adherent. The most common reasons for non-adherence were forgetting to take the medication or lacking knowledge about the prescribed medication. Multiple linear regression revealed that adherence was good in people with external HLC and poor in internal HLC. In particular, intentional non-adherence was positively associated with internal HLC and negatively with fatalistic external HLC. Depression had a negative influence on both intentional and unintentional non-adherence.

**Conclusion:** HLC is an independent predictor of medication non-adherence and is a promising target for interventions that enhance adherence.

## Introduction

Health locus of control (HLC) refers to whether individuals think they can take responsibility for their health or not. It reflects the degree to which someone attributes the consequences of their behavior to internal or external factors. The original concept was developed in 1954 by Julian B. Rotter in his Social Learning Theory (1) and expanded by Wallston et al. (2). HLC is considered internal (a belief that one can control one's health) or external (a belief that external factors control health). External factors are external social control (control by other persons, physicians) and fatalistic external control (control by fate, god, chance). Therefore, people with a high internal HLC should be more likely to engage in health-promoting and -sustaining activities. In contrast, people who believe that their health is beyond their control are probably less likely to implement recommended health behavior (3).

HLC also plays an important role in medication adherence (3). Adherence describes the extent to which patients take their medication as agreed with their health care providers (4). Patient-related factors, condition-related factors, and therapy-related factors influence adherence (5, 6). Non-adherence is common in patients with chronic disorders and contributes to higher morbidity, higher mortality, and increased healthcare costs. Non-adherence can be intentional (e.g., if a patient deliberately decides not to take medication) or unintentional (e.g., forgetting to take medication) (7).

However, HLC's contribution to non-adherence in general, and intentional or unintentional non-adherence, needs clarification. One study of women on hormone therapy reported that patients who deliberately missed their medication viewed themselves as having less influence over their health than patients who reported forgetting their medication. Moreover, women who reported forgetting their medication also had a stronger belief that powerful others (such as health professionals, family, friends) are relevant to improving their health than patients who intentionally chose not to take their hormones (8). However, this study's results cannot be generalized; therefore, we aimed to explore the relationship between HLC and unintentional/intentional non-adherence in a community-dwelling population. We hypothesized that internal HLC is associated with intentional non-adherence and that external HLC is associated with less intentional non-adherence.

## Methods

### Study Design and Participants

The ethics committee of Jena University Hospital (5290-10/17) and the Landesärztekammer Thüringen approved the study. In this cross-sectional study, several questionnaires were distributed to three randomly selected family doctors' practices in Jena, Germany, between September 2019 and December 2019. Patients completed the questionnaires anonymously in the waiting room and placed them in a box. Of the 200 questionnaires distributed, 188 were completed. The inclusion criteria were (1) age over 18, (2) at least one permanent medication, and (3) the absence of dementia according to medical records, and (4) the ability to fill out the questionnaires independently.

### Questionnaires

The self-reported demographic data included age, gender, marital status (single, divorced/widowed or married), and level of education (high: German *Abitur* or university; medium: German *Realschule* or General Certificate of Secondary Education; low: German *Hauptschule* or no school). Additionally, patients were asked about their diseases and the total daily number of medications administered. Adherence was assessed using the German Stendal adherence to medication score (SAMS) (9), which is based on 18 items forming a cumulative scale (0–72), where 0 represents complete adherence and 72 complete non-adherence (10). The SAMS allows assessment of three common patterns or reasons for non-adherence: modification of medications, forgetting to take the medications, and lacking knowledge about the medications (7). The entire SAMS and the manual are available online (CC BY NC 3.0 license; https://data.mendeley.com/datasets/ny2krr3vgg/1) (9, 11).

To examine the patients' perceptions of their autonomy support through the doctor and assess the patient-physician relationship, the German version of the Health Care Climate Questionnaire (HCCQ) was used. Autonomy support consists of providing patients with effective treatment options, supporting patient initiatives, eliciting and considering patients' views, and minimizing control and judgment. The HCCQ consists of 15 items on a seven-point Likert scale ranging from 1 = strongly disagree to 7 = strongly agree. Only item 13 is reverse-coded. The individual score is calculated with the mean score after reversing item 13. Higher mean scores indicate a higher level of perceived autonomy support (12).

To determine the patients' HLC, we used the German questionnaire “Kontrollüberzeugung zu Krankheit und Gesundheit” (KKG), which assessed the overall control belief for disease and health. It is based on the Multidimensional HLC Scale (MHLC). Its 21 items are statements with a six-point Likert scale from “Does not apply at all” (value 1) to “Very much applies” (value 6). From the item response, a cumulative score is formed for three subscales (from min. 7 to max. 42): internal, social external, and fatalistic external HLC. High sum values indicate high HLC and low sum values low HLC. Internality represents the belief in their self-control of health-related events. Social externality means that a person thinks that their physical condition depends largely on the actions of others (e.g., physicians). A fatalistic externality is a conviction that fate and chance influence their state of health.

The Patient Health Questionnaire (PHQ)-9 questionnaire was used to detect depression. Its 9 items cover DSM-IV criteria for the diagnosis of depression. The cumulative score can range from 0 to 27 and assigns patients to the categories “no depression” (<10), “mild depression” (10–19), and “manifest depression” (20–27) (13, 14).

Due to severely incomplete or uncompleted questionnaires (*n* = 14) and contradictory response behavior (*n* = 14), 28 patients were excluded from the analysis. Excluded patients tended to be younger (median = 59, IQR = 25.75), most had a high educational level (*N* = 13, 46.5%), and took an average of 3 medications per day (IQR = 2). The number of drugs per day in the excluded patients did not significantly differ from the included patients (median = 4, IQR = 4; *p* = 0.23). The central tendency of the KKG and HCCQ questionnaire responses did not differ from the included patients. The median sum score of the PHQ-9 was 6.0 (IQR = 5.25) in the excluded patients, slightly higher than in the included patients (median = 4.0, IQR = 6.0).

### Statistics

SPSS version 23.0 (IBM Corporation, Armonk, NY, USA) and R statistics version 4.0.2 (R Foundation for Statistical Computing, Vienna, Austria were used for the statistical analyses. Descriptive analyses were used to describe the cohort. The cut-off for clinically significant non-adherence was set at a total SAMS of 9 points (80th percentile). Generally, non-adherence becomes clinically significant when <80% of a prescribed medication is taken (15, 16). The cohort was categorized study and sample-specific into adherent (SAMS = 0), moderately non-adherent (SAMS = 1–8), and non-adherent (SAMS ≥ 9) patients. Patients were categorized according to the main reason/factor of non-adherence: medication modifications, missing knowledge about medication, and forgetfulness (7, 9). For this purpose, the items that belonged to a factor were summed and divided by the number of items resulting in three SAMS factors. In the factor' modifications', medications were changed by the patients without consulting their doctors about either experiencing side effects or health improvement. The factor' missing knowledge' included patients who were unaware of the purpose and/or dosage of their medication. The factor' forgetfulness' included patients who forgot their medication frequently.

In addition, patients were categorized into three HLC groups: internal, social external, and fatalistic external HLC. The groups' clinical and sociodemographic parameters were compared by analysis of variance, the Kruskal–Wallis test, and the chi-square test. Four linear regressions were performed with the (1) SAMS total, (2) the SAMS modification factor, (3) the SAMS missing knowledge factor, and (4) the SAMS forgetting factor as the dependent variables and age, gender, education level, number of medications per day, internal HLC subscore, social external HLC subscore, fatalistic external HLC subscore, the HCCQ average score, and the PHQ9 sum score as independent variables (backward selection). The significance levels for variables entering the linear regression model and for removing from the model were set at 0.05 and 0.1, respectively. Autocorrelation (Durbin–Watson) and multicollinearity (variance inflation factor and tolerance) were excluded. However, residuals were not normally distributed for the models with the SAMS factors as dependent variables. Although the multiple linear regression is mostly robust to violations of the normal distribution assumption, we computed additional elastic net models to test the results (17). Elastic net regularization leads to parsimonious models, which are easier to interpret. Variable selection is performed by shrinking the parameters toward zero and attenuating overfitting. Ten-fold cross validation was performed to choose the best model with the lowest mean cross-validated error. Within the elastic net algorithm, variables remain in the model if the prediction error averaged over the ten-fold cross validation samples is reduced. The elastic net algorithm performs well in highly correlated variables, either including all variables with similar regression coefficients or excluding all variable from the best model. Elastic net regularization was performed using the package glmnet in R version 3.6.2 (R Foundation for Statistical Computing, Vienna, Austria). Again using elastic net, four models were calculated with the (1) SAMS total, (2) the SAMS modification factor, (3) the SAMS missing knowledge factor, and (4) the SAMS forgetting factor as the dependent variables and age, gender, educational level, number of medications per day, internal HLC subscore, social external HLC subscore, fatalistic external HLC subscore, the HCCQ average score, and the PHQ9 sum score as independent variables.

Normally distributed values are expressed as means and standard deviation (SD); other values are expressed as medians and interquartile range (IQR). All categorical variables are presented as numbers and percentages. Statistical significance was set at a *p*-value < 0.05.

## Results

### Descriptive Statistics

Data from 160 patients were used for the following analyses. Most patients were 60 years or older (65%), female, married, pensioned, and had a middle or higher education level (Table 1). The self-reported diseases comprised a wide spectrum; hypertension, diabetes, and thyroid diseases were the most common. The number of medications taken daily was 4.4 (SD = 3.0) (Table 1). According to the PHQ-9, 123 (78.8%) reported no, 24 (15.4%) reported mild, 6 (3.8%) moderate, and 3 (1.9%) severe depression (4 missing data). The HCCQ indicated that the patient's perceived support for autonomy through the doctor was very high.

Table 1Characteristics of the cohort.

**Table:** 

**Characteristic**	***n***	**%**	**Missing**
**Gender**			
Female	98	61.3	0
Male	62	38.8	
**Education**			
Low	32	20.0	0
Middle	65	40.6	
High	63	39.4	
**Marital status**			
Not married	54	34.2	2
Married	104	65.8

**Table:** 

	**Mean**	**SD**	**Median**	**IQR**	**Missing**
Age	63.3	14.9	67.0	23	0
SAMS sum score	4.3	4.9	3	4.9	0
HCCQ mean value	6.3	1.0	6.6	0.73	27
PHQ-9 sum score	5.7	4.8	4	6	4
Subtotal score internal HLC	24.94	6.148	26	8	12
Subtotal score social external HLC	23.89	5.209	24	7.25	18
Subtotal score fatalistic external HLC	18.59	6.684	18	10	20

*SAMS, Stendal adherence to medication score; HCCQ, Health Care Climate Questionnaire; PHQ-9, Patient Health Questionnaire-9; HLC, Health locus of control*.

### Adherence

**Figure 1:**
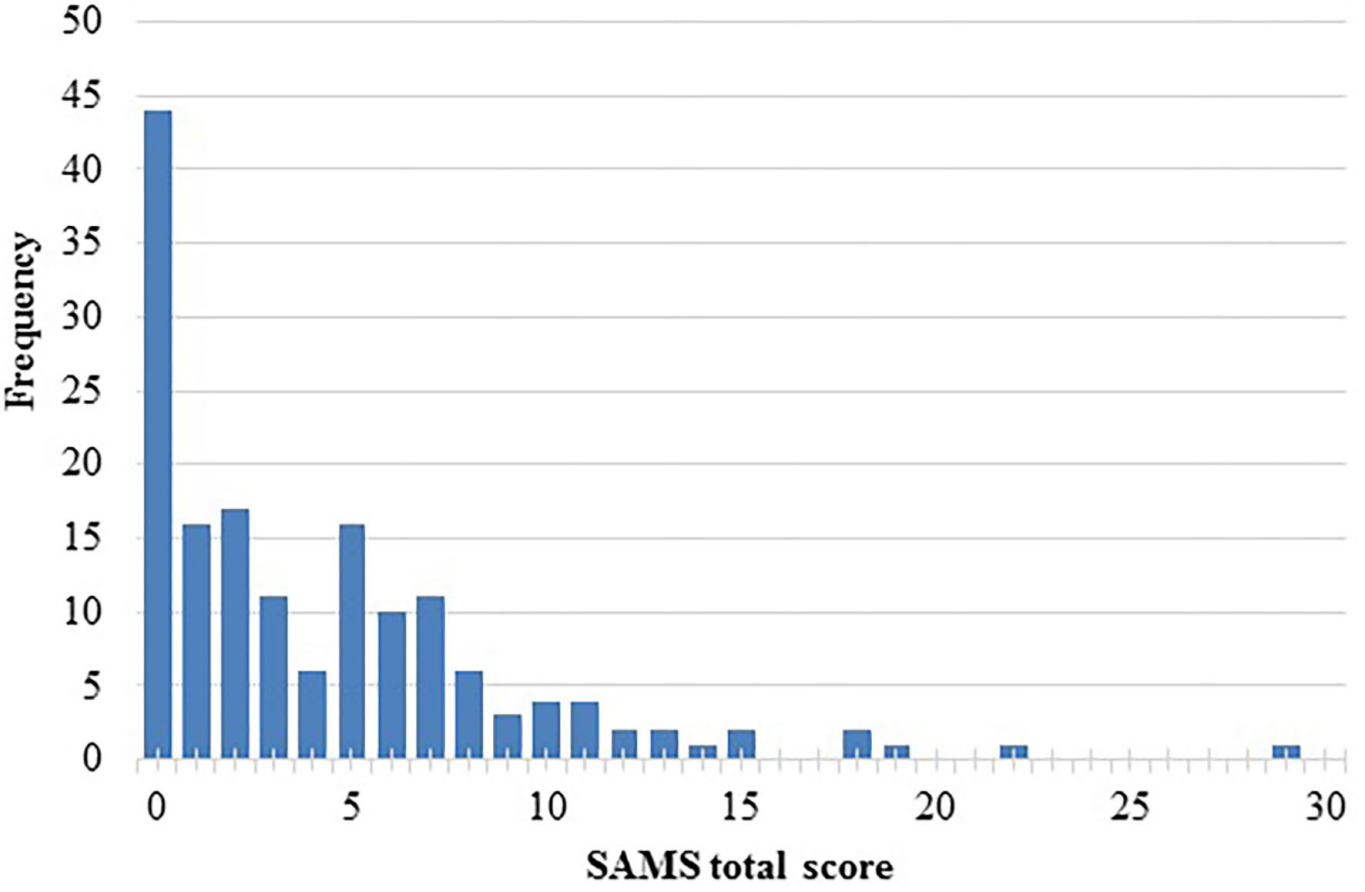
Distribution of Stendal Adherence to Medication Score (SAMS) total score.

According to the SAMS, 44 (27.5%) reported being fully adherent (SAMS = 0), 93 (58.1%) reported moderate non-adherence (SAMS = 1–8), and 23 (14.4%) clinically significant non-adherence (SAMS ≥ 9). Figure 1 displays the SAMS distribution. The main reasons for non-adherence were forgetting (*n* = 55, 47.4%), missing knowledge about prescribed medication (*n* = 39, 33.6%), and modifying prescribed medication (*n* = 22, 19.0%) (refers to the 116 participants with a SAMS > 1). The partial correlation between the SAMS total score and the PHQ-9 score, adjusted for the influence of the variable age, was positive with an *r* = 0.201 (*p* = 0.012).

### HLC Beliefs

Table 1 lists the mean sum scores of the three HLC subscales. The highest subscale was determined for every patient; consequently, 30 (18.8%) participants had predominantly fatalistic external HLC, 70 (43.8%) social external HLC, and 11 (6.9%) internal HLC. Furthermore, 28 (17.5%) overlapped with equal values for 2 subscales; 21 subjects could not be categorized due to missing data (a total of 139 analyzed KKG questionnaires).

The three HLC groups did not differ in PHQ-9 (*p* = 0.20), HCCQ (*p* = 0.79), sex (*p* = 0.13), and marital status (*p* = 0.09) (Table 2). Patients with internal HLC were younger than patients with external social HLC (*p* = 0.024). Age showed a weak negative correlation with internal HLC (*r* = −0.265, *p* = 0.01). Age did not correlate with social external or fatalistic external HLC.

Table 2Characteristics of people with predominantly internal, social external, and fatalistic external health locus of control.

**Table:** 

**Characteristic**	**Internal health locus of control**		**Social-external health locus of control**		**Fatalistic-external health locus of control**	
	***n***	**%**	***n***	**%**	***n***	**%**
**Gender**						
Female	9	81.8	36	51.4	19	63.3
Male	2	18.2	34	48.6	11	36.7
**Education**						
Low	1	9.1	9	12.9	8	26.7
Middle	6	54.5	23	32.9	16	53.3
High	4	36.4	38	54.3	6	20.0
**Marital status**						
Not married	2	20.0	18	26.1	14	46.7
Married	8	80.0	51	73.9	16	53.3

**Table:** 

	**Mean**	**95% CI**		**Mean**	**95% CI**		**Mean**	**95% CI**	
Age	52.45	44.32	60.59	65.19	61.79	68.58	63.53	57.65	69.42
SAMS sum score	6.36	0.52	12.21	4.47	3.33	5.62	4.27	2.30	6.24
HCCQ mean value	6.43	6.02	6.83	6.21	5.94	6.48	6.17	5.66	6.67
PHQ-9 sum score	5.82	1.93	9.71	5.14	4.14	6.15	7.03	4.91	9.15
Subscore internal HLC	32.09	30.38	33.80	24.51	23.04	25.98	23.40	21.50	25.30
Subscore social external HLC	19.70	18.01	21.39	26.76	25.68	27.83	20.45	18.64	22.25
Subscore fatalistic external HLC	13.00	9.78	16.22	16.70	15.23	18.16	25.40	23.76	27.04

*SAMS, Stendal adherence to medication score; HCCQ, Health Care Climate Questionnaire; PHQ-9, Patient Health Questionnaire-9; HLC, Health locus of control*.

### Adherence and HLC Beliefs

The overall adherence (SAMS total score) did not differ between the three HLC groups (*p* = 0.52) (Table 2). The SAMS did not correlate with the sum scores of the three HLC domains. Vice versa, the sum scores of the HLC domains did not differ between adherent (SAMS = 0), moderately non-adherent (SAMS 1 to 8), and clinically significant non-adherent patients (SAMS ≥ 9) (Table 3).

Table 3Characteristics of people with different degrees of non-adherence.

**Table:** 

**Characteristic**	**Adherent**		**Moderately non-adherent**		**Non-adherent**	
	***n***	**%**	***n***	**%**	***n***	**%**
**Gender**						
Female	30	68.2	53	57.0	15	65.2
Male	14	31.8	40	43.0	8	34.8
**Education**						
Low	11	25.0	19	20.4	2	8.7
Middle	21	47.7	32	34.4	12	52.2
High	12	27.3	42	45.2	9	39.1
**Marital status**						
Not married	19	45.2	29	31.2	6	26.1
Married	23	54.8	64	68.8	17	73.9

**Table:** 

	**Mean**	**95% CI**		**Mean**	**95% CI**		**Mean**	**95% CI**	
Age	66.16	62.25	70.07	63.61	60.54	66.68	56.83	49.48	64.17
SAMS sum score	0.00	0.00	0.00	4.00	3.53	4.47	13.52	11.40	15.64
HCCQ mean value	6.39	6.07	6.70	6.19	5.95	6.42	6.38	6.17	6.59
PHQ-9 sum score	5.21	3.60	6.82	5.53	4.58	6.49	7.26	5.34	9.18
Subscore internal HLC	23.18	20.70	25.66	25.43	24.22	26.64	26.09	24.13	28.04
Subscore social-external HLC	22.61	20.77	24.44	24.37	23.23	25.51	24.30	22.32	26.29
Subscore fatalistic-external HLC	18.87	16.53	21.21	18.29	16.84	19.73	19.23	16.18	22.28

*SAMS, Stendal adherence to medication score; HCCQ, Health Care Climate Questionnaire; PHQ-9, Patient Health Questionnaire-9; HLC, Health locus of control*.

### Multiple Linear Regression

Regression analyses examined the influence of clinical variables on (1) SAMS total, (2) factor SAMS modification, (3) factor SAMS knowledge, and (4) factor SAMS forgetting. Age, gender, education level, number of medications per day, internal HLC subscore, social external HLC subscore, fatalistic external HLC subscore, the HCCQ average score, and the PHQ9 sum score were entered as independent variables (backward selection).

The PHQ-9, internal HLC, and fatalistic external HLC predicted the total SAMS total score (the final models after backward selection are given in Table 4, the detailed models are given as Supplementary Tables 1–4). Depression and internal HLC were associated with non-adherence. Fatalistic external HLC was associated with better adherence (Supplementary Table 1). However, the explained variance was low. The influence of depression and HLC on the SAMS total score was also demonstrated in the elastic net model (Supplementary Table 5). Overall, the strongest influence had depression on the SAMS total.

**Table 4:** Summary of multiple linear regressions (final models after backward selection).

	**Std. coefficient B**	**Std. Err**	***p***	**Overall model**
**SAMS total (dependent variable)**				
Subtotal score internal HLC	0.146	0.080	0.072	*p* 0.003 corrected R^2^ 0.096
Subtotal score fatalistic external HLC	−0.132	0.078	0.093	
PHQ-9 sum score	0.339	0.101	0.001	
**SAMS modification (dependent variable)**				
Subtotal score internal HLC	0.016	0.008	0.053	*p* 0.012 corrected R^2^ 0.076
Subtotal score fatalistic external HLC	−0.016	0.008	0.037	
PHQ-9 sum score	0.025	0.010	0.012	
**SAMS missing knowledge (dependent variable**)				
Education level (1 = low, 2 = middle, 3 = high)	−0.109	0.052	0.038	*p* 0.007 corrected R^2^ 0.094
Number of medications per day	0.028	0.013	0.031	
Subtotal score fatalistic external HLC	−0.013	0.006	0.027	
PHQ-9 sum score	0.016	0.008	0.049	
**SAMS forgetting (dependent variable)**				
Gender (0= female, 1= male)	0.216	0.109	0.052	*p* 0.025 corrected R^2^ 0.080
Age	−0.008	0.003	0.017	

*SAMS, Stendal adherence to medication score; PHQ-9, Patient Health Questionnaire-9; HLC, Health locus of control*.

*SAMS modification* was positively associated with internal HLC and depression and negatively associated with fatalistic external HLC. This finding indicates external HLC is related to less intentional non-adherence (Table 4, Supplementary Table 2). The elastic net model confirmed the influence of depression on the *SAMS modification* (Supplementary Table 6). In addition lower age was associated with more modification of medication. However, HLC was not a relevant predictor in the elastic net model. Considering the two models with low coefficients and low explained variance, one can conclude that HLC plays only a minor role for self-reported intentional non-adherence/modification.

The SAMS *missing knowledge* was associated with depression, lower educational level, a higher number of drugs per day, and fatalistic external HLC (Table 4, Supplementary Table 3). The relevance of fatalistic external HLC and the number of drugs per day on the self-reported knowledge about medication could also be confirmed in the elastic net model (Supplementary Table 7). To a lesser extent, gender, depression, and education level were also relevant factors for SAMS *missing knowledge* here.

*Forgetting to take medication* was more likely reported in young and male persons (Table 4, Supplementary Table 4). This was in line with the findings in the elastic net model (Supplementary Table 8). However, the explained variance was low.

## Discussion

This cross-sectional study aimed to analyze the frequency and reasons for non-adherence in patients from general practices and explore the relationship between HLC and unintentional/intentional non-adherence. We hypothesized that internal HLC is associated with intentional non-adherence. Furthermore, we assumed that external HLC is associated with less intentional non-adherence.

In our cohort, about three-quarters (72.5%) reported different degrees of non-adherence. Most were moderately non-adherent, and only a minority were clinically significant non-adherent. The reasons for non-adherence were mostly unintentional (e.g., forgetting to take medication, 47.4%). The prevalence of non-adherence strongly depends on the method of measurement (objective or subjective). Various reviews reported non-adherence ranging from 20 to 80% of cases for certain chronic diseases, especially hypertension and diabetes (5, 18, 19). Our findings are consistent with previous studies showing that *forgetting* is the primary self-reported behavior identified among non-adherent participants (20). In this survey, *forgetting to take medication* was the most frequent reason for non-adherence, followed by a *lack of knowledge* about the prescribed medication and *modification of medication*.

In our study, we also assessed the patients perceived autonomy support through the doctor using the HCCQ. The mean score was very high, implying that patients feel strongly supported by their family doctor. Another reason for the high mean value of HCCQ might be the traditionally close relationship between general practitioners and patients in Germany, which might discourage patients from criticizing their family doctor (with whom they have a long-standing relationship). But how does the doctor-patient relationship affect adherence? With correction for demographic factors, depression, and HLC, we did not observe a relevant association between overall adherence (SAMS total) and HCCQ. On the one hand, a trusting relationship could ensure that patients do not modify the prescribed medication regimen and are less intentionally non-adherent. On the other hand, patients might feel uncomfortable reporting medication modification because of this special relationship between the patient and the general practitioner. This is why we used the anonymous design of the study.

In the DEGS-1 study in Germany, between 2008 and 2011, almost 8,000 people were surveyed with the PHQ-9 questionnaire about current depressive symptoms using the same cut-off values as we did. According to self-disclosure, approximately 8% of the German population between 18 and 79 suffer from depressive symptoms (21). In our cohort, 21.2% of the respondents reported depressive symptoms, which is higher than the general prevalence in Germany. Depressive symptoms impair adherence (22), as indicated in our study with a weak effect strength. However, patients with depressive symptoms might be more self-critical when reporting their adherence (22).

HLC was assessed by the German KKG questionnaire. The KKG is a modification of the “multidimensional HLC” (MHLC) (2) questionnaire. It is based on the same theoretical and methodological concept. Still, the KKG has a unique aspect in the formulation of its items: Patients are asked about their perceived ability to influence their physical *conditions* (physical complaints, physical well-being). In contrast, the MHLC uses the terms *illness* and *health*. Yet, chronically ill patients might find it difficult to use the term *health* referring to their situation. Therefore, the rather neutral terms of the KKG questionnaire are more suited for heterogeneous patient populations, as they are found in general practices. Most patients in our survey had an external social control belief. The external social HLC includes all persons who influence his or her physical condition according to the respondent's perception. Therefore, all that can be said is that a high proportion of patients in our study believe that other people strongly influence their physical condition.

There are conflicting results in terms of the relationship between HLC and adherence. Nafradi et al. (3) published a review of 154 studies on the relationship between self-efficacy, HLC, and adherence. Of these, 71 studies refer to the relationship between HLC and adherence. The studies applying subjective adherence measures reported more association either in a positive or negative direction (58.5%) between the HLC dimensions and adherence compared to the studies applying mixed (14.3%) or objective (40%) adherence measures. More than half of the studies report no link between HLC and medication adherence. Doctors' HLC was most likely to show a positive association with adherence (3).

Regarding the link between internal HLC and adherence, which is considered the most predictive HLC dimension in the literature, only 10 studies out of 26 (38%) showed a positive association; 15 studies (58%) showed no association. We found that adherence was better in people with external HLC and worse in people with internal HLC. In particular, *modification of prescribed drugs without physician consultation* was positively associated with internal HLC and negatively with fatalistic external HLC. However, one has to keep in mind that the explained variance in the model was low. This indicates that HLC plays a role for adherence, but its influence should not be overestimated.

Some methodological aspects clarify these findings. The German KKG questionnaire that we used to measure HLC is a modification of the MHCL questionnaire by Wallston et al. (2). However, Wallston himself questioned HLC as a predictor of health behavior (23). In a summary of several studies using his MHCL questionnaire, he described that the loci of control could explain on average <10% of the variance of specific health behavior (23). *Locus of control* is a generalized expectancy, a belief that one can control one's health. It is a kind of personality trait. In contrast, *self-efficacy* is the belief that one can manage a certain situation or behavior. It is state-related instead of trait-related. It is possible to have an internal locus of control regarding one's health. Still, if the conviction in one's capabilities to carry out a specific behavior is missing (as in adherence to a certain medication), one is not likely to engage in that behavior. In this example, there could be a high level of internal HLC and simultaneously low self-efficacy regarding medication adherence (23). Medication adherence means the reliable and correct intake of prescribed medication as agreed by doctor and patient. It is a situational health behavior. Therefore, we suggest that in the context of adherence research, the situation-specific self-efficacy belief should be studied in addition to HLC. Future studies incorporating the locus of control concept should also ask how important adherence and the resulting better outcome are to the respondents.

Moreover, for further studies, it would be interesting to study the relationship between adherence and HLC after subdividing external social HLC into 1. physicians and 2. other influencing persons (24). This relationship could also help to understand the influence of this scale on adherence. The review mentioned above on HLC and adherence by Nafradi et al. also reported that physicians tend to influence adherence (3) positively. Other people, such as family and friends, have an ambivalent influence, depending on whether these others support or discourage the patients to adhere (3). Future research should divide external social HLC into two subgroups so that a total of four dimensions of locus of control can be assessed; i.e., internality, formal help (=physicians), informal help (=other people), and chance to investigate these beliefs appropriately. Furthermore, the formal help dimension would need a broader assessment since it is covered by only two items (24).

Our study has certain limitations. The cross-sectional design does not allow causal statements. The study design creates the possibility of self-report bias in terms of socially desirable answers. We aimed to reduce this bias by using an anonymous design of the study. In addition, the HCCQ questionnaire showed limited informative value due to the very high mean values and their reduced variability. Only patient-related predictors of non-adherence were investigated in our study. In addition, however, there are various other reasons for non-adherence that we did not record with the anonymous design. Thus, there is a risk of presenting the complex construct of non-adherence in a simplified way. Although it is widely accepted and common to use a threshold of 80% to determine non-adherence, this approach remains arbitrary when there is no objective measure of adherence or other measurable clinical outcome. Therefore, we incorporated group comparisons and correlation analyses in our study to overcome the problem of arbitrary adherence cut-offs. Finally, we cannot make precise statements about the cognition of the patients who participated in our study. The practice staff was asked to distribute the questionnaires according to our inclusion criteria, but this cannot be verified afterward in an anonymous study. Also an analysis taking underlying main disorders and disease durations as well as social networks into account would be a promising approach to study the interaction between adherence and HLC in detail.

## Conclusions

The complex reasons for non-adherence will not be measured or represented in their entirety in any study and cannot be assessed with single questionnaires. It is helpful to obtain and evaluate empirical data on adherence. However, equally important is the willingness in a trusting doctor-patient relationship to work together on an individualized concept of illness and recovery. In this context, patient decision-making also requires adequate health literacy, and not every patient wants to be transferred this competence. Further research is needed to find the right amount of control adjusted to different physical conditions and stages of the disease. For this, qualitative research could also play an important role, as it manages to go into depth and capture complexity by interviewing individual cases.

## Data Availability Statement

The raw data supporting the conclusions of this article will be made available by the authors, without undue reservation.

## Ethics Statement

The studies involving human participants were reviewed and approved by ethics committee of Jena University Hospital (5290-10/17) and the Landesärztekammer Thüringen.

## Author Contributions

H-MG: data acquisition, statistical analyses, and writing first draft. TP: study concept, review and editing. All authors contributed to the article and approved the submitted version.

## Conflict of Interest

The authors declare that the research was conducted in the absence of any commercial or financial relationships that could be construed as a potential conflict of interest.

## Publisher's Note

All claims expressed in this article are solely those of the authors and do not necessarily represent those of their affiliated organizations, or those of the publisher, the editors and the reviewers. Any product that may be evaluated in this article, or claim that may be made by its manufacturer, is not guaranteed or endorsed by the publisher.
