# Supplementary material for: Association Between the Health Locus of Control and Medication Adherence: An Observational, Cross-Sectional Study in Primary Care
Source: Front Med (Lausanne). 2021 Aug 16;8:705202. doi: 10.3389/fmed.2021.705202 (PMC8418132; doi:10.3389/fmed.2021.705202)
Supplement: Supplementary file 1 [file Data_Sheet_1.docx]

**Supplement**

[Table 1. Coefficients of linear regression with SAMS total as dependent variable 2](#_Toc77712429)

[Table 2. Coefficients of linear regression with SAMS modification as dependent variable 4](#_Toc77712430)

[Table 3. Coefficients of linear regression with SAMS missing knowledge as dependent variable 6](#_Toc77712431)

[Table 4. Coefficients of linear regression with SAMS forgetting as dependent variable 8](#_Toc77712432)

[Table 5. Elastic net model with SAMS total as dependent variable. 10](#_Toc77712433)

[Table 6. Elastic net model with SAMS modification as dependent variable. 11](#_Toc77712434)

[Table 7. Elastic net model with SAMS missing knowledge as dependent variable. 12](#_Toc77712435)

[Table 8. Elastic net model with SAMS forgetting as dependent variable. 13](#_Toc77712436)

# Table 1. Coefficients of linear regression with SAMS total as dependent variable

| Model | | Unstandardized Coefficients | |  | t | Sig. | 95% CI | |
| --- | --- | --- | --- | --- | --- | --- | --- | --- |
|  |  | B | Std. Error | Std. coefficient B |  |  | Lower Bound | Upper Bound |
| 1 | (Constant) | 3.198 | 5.375 |  | .595 | .553 | -7.468 | 13.864 |
|  | Gender | -.022 | 1.154 | -.002 | -.019 | .985 | -2.313 | 2.268 |
|  | Age | -.053 | .042 | -.148 | -1.274 | .206 | -.136 | .030 |
|  | Education | .055 | .717 | .008 | .076 | .939 | -1.368 | 1.477 |
|  | Number of drugs per day | .104 | .200 | .059 | .523 | .602 | -.292 | .500 |
|  | internal HLC | .117 | .092 | .137 | 1.272 | .206 | -.065 | .299 |
|  | social-external HLC | .024 | .115 | .023 | .210 | .834 | -.204 | .253 |
|  | fatalistic-  external HLC | -.135 | .086 | -.167 | -1.575 | .118 | -.305 | .035 |
|  | HCCQ mean value | .196 | .531 | .036 | .370 | .712 | -.857 | 1.250 |
|  | PHQ-9 sum score | .302 | .110 | .281 | 2.748 | .007 | .084 | .520 |
|  | Model 1 Corrected R ^2^: .061 | | | | | | | |
| 2 | (Constant) | 3.202 | 5.346 |  | .599 | .551 | -7.405 | 13.808 |
|  | Age | -.053 | .041 | -.149 | -1.314 | .192 | -.134 | .027 |
|  | Education | .054 | .712 | .007 | .076 | .940 | -1.359 | 1.466 |
|  | Number of drugs per day | .104 | .197 | .059 | .527 | .599 | -.287 | .495 |
|  | internal HLC | .117 | .091 | .137 | 1.278 | .204 | -.065 | .298 |
|  | social-external HLC | .024 | .113 | .023 | .211 | .833 | -.200 | .248 |
|  | fatalistic-  external HLC | -.135 | .085 | -.167 | -1.584 | .116 | -.304 | .034 |
|  | HCCQ mean value | .198 | .523 | .036 | .378 | .706 | -.841 | 1.236 |
|  | PHQ-9 sum score | .302 | .108 | .282 | 2.807 | .006 | .089 | .516 |
|  | Model 2 Corrected R^2^: .071 | | | | | | | |
| 3 | (Constant) | 3.328 | 5.052 |  | .659 | .511 | -6.693 | 13.349 |
|  | Age | -.054 | .040 | -.150 | -1.331 | .186 | -.134 | .026 |
|  | Number of drugs per day | .103 | .196 | .058 | .526 | .600 | -.286 | .492 |
|  | internal HLC | .117 | .091 | .137 | 1.282 | .203 | -.064 | .297 |
|  | social-external HLC | .026 | .108 | .025 | .241 | .810 | -.189 | .241 |
|  | fatalistic-  external HLC | -.136 | .083 | -.168 | -1.640 | .104 | -.301 | .029 |
|  | HCCQ mean value | .196 | .520 | .036 | .377 | .707 | -.836 | 1.229 |
|  | PHQ-9 sum score | .302 | .107 | .281 | 2.821 | .006 | .090 | .514 |
|  | Model 3 Corrected R^2^:.080 | | | | | | | |
| 4 | (Constant) | 3.633 | 4.867 |  | .746 | .457 | -6.021 | 13.288 |
|  | Age | -.054 | .040 | -.150 | -1.339 | .183 | -.133 | .026 |
|  | Number of drugs per day | .111 | .192 | .063 | .576 | .566 | -.271 | .492 |
|  | internal HLC | .124 | .084 | .146 | 1.478 | .143 | -.043 | .292 |
|  | fatalistic-  external HLC | -.133 | .082 | -.164 | -1.630 | .106 | -.295 | .029 |
|  | HCCQ mean value | .203 | .517 | .037 | .393 | .695 | -.823 | 1.229 |
|  | PHQ-9 sum score | .300 | .106 | .280 | 2.824 | .006 | .089 | .511 |
|  | Model 4 Corrected R^2^: .088 | | | | | | | |
| 5 | (Constant) | 4.927 | 3.569 |  | 1.381 | .170 | -2.151 | 12.006 |
|  | Age | -.055 | .040 | -.155 | -1.396 | .166 | -.134 | .023 |
|  | Number of drugs per day | .106 | .191 | .060 | .553 | .581 | -.273 | .485 |
|  | internal HLC | .125 | .084 | .147 | 1.493 | .138 | -.041 | .292 |
|  | fatalistic-  external HLC | -.127 | .080 | -.157 | -1.592 | .114 | -.286 | .031 |
|  | PHQ-9 sum score | .297 | .106 | .277 | 2.814 | .006 | .088 | .506 |
|  | Model 5 Corrected R^2^: .096 | | | | | | | |
| 6 | (Constant) | 4.758 | 3.544 |  | 1.343 | .182 | -2.270 | 11.786 |
|  | Age | -.045 | .035 | -.127 | -1.289 | .200 | -.115 | .024 |
|  | internal HLC | .118 | .083 | .139 | 1.431 | .155 | -.046 | .282 |
|  | fatalistic-  external HLC | -.119 | .078 | -.147 | -1.522 | .131 | -.275 | .036 |
|  | PHQ-9 sum score | .299 | .105 | .279 | 2.847 | .005 | .091 | .507 |
|  | Model 6 Corrected R^2^: .102 | | | | | | | |
| 7 | (Constant) | 1.229 | 2.258 |  | .544 | .587 | -3.248 | 5.706 |
|  | internal HLC | .146 | .080 | .171 | 1.818 | .072 | -.013 | .305 |
|  | fatalistic-  external HLC | -.132 | .078 | -.163 | -1.696 | .093 | -.287 | .022 |
|  | PHQ-9 sum score | .339 | .101 | .316 | 3.361 | .001 | .139 | .539 |
|  | Model 7 Corrected R^2^: .096 | | | | | | | |
|  | | | | | | | | |

# Table 2. Coefficients of linear regression with SAMS modification as dependent variable

| Model | | Unstandardized Coefficients | |  | t | Sig. | 95% CI | |
| --- | --- | --- | --- | --- | --- | --- | --- | --- |
|  |  | B | Std. Error | Std. coefficient B |  |  | Lower Bound | Upper Bound |
| 1 | (Constant) | .062 | .532 |  | .117 | .907 | -.993 | 1.118 |
|  | Gender | -.021 | .112 | -.021 | -.191 | .849 | -.244 | .202 |
|  | Age | -.003 | .004 | -.076 | -.634 | .527 | -.011 | .006 |
|  | Education | .085 | .070 | .124 | 1.218 | .226 | -.053 | .223 |
|  | Number of drugs per day | .000 | .019 | -.001 | -.008 | .994 | -.039 | .039 |
|  | internal HLC | .014 | .009 | .171 | 1.519 | .132 | -.004 | .033 |
|  | social-external HLC | -.004 | .011 | -.043 | -.382 | .703 | -.026 | .018 |
|  | fatalistic-external HLC | -.013 | .009 | -.162 | -1.472 | .144 | -.030 | .004 |
|  | HCCQ mean value | -.009 | .051 | -.018 | -.179 | .858 | -.111 | .092 |
|  | PHQ-9 sum score | .022 | .011 | .222 | 2.117 | .037 | .001 | .044 |
|  | Model 1 Corrected R^2^: .042 | | | | | | | |
| 2 | (Constant) | .062 | .529 |  | .118 | .906 | -.987 | 1.112 |
|  | Gender | -.022 | .111 | -.021 | -.194 | .847 | -.242 | .199 |
|  | Age | -.003 | .004 | -.077 | -.692 | .491 | -.010 | .005 |
|  | Education | .085 | .069 | .124 | 1.229 | .222 | -.052 | .222 |
|  | internal HLC | .014 | .009 | .171 | 1.558 | .122 | -.004 | .032 |
|  | social-external HLC | -.004 | .011 | -.043 | -.390 | .698 | -.026 | .017 |
|  | fatalistic-external HLC | -.013 | .008 | -.162 | -1.504 | .136 | -.029 | .004 |
|  | HCCQ mean value | -.009 | .051 | -.018 | -.180 | .858 | -.110 | .092 |
|  | PHQ-9 sum score | .022 | .011 | .222 | 2.131 | .036 | .002 | .043 |
|  | Model 2 Corrected R^2^: .052 | | | | | | | |
| 3 | (Constant) | .005 | .421 |  | .012 | .990 | -.830 | .840 |
|  | Gender | -.019 | .109 | -.018 | -.171 | .864 | -.236 | .198 |
|  | Age | -.003 | .004 | -.074 | -.679 | .499 | -.010 | .005 |
|  | Education | .085 | .069 | .125 | 1.241 | .217 | -.051 | .221 |
|  | internal HLC | .014 | .009 | .171 | 1.564 | .121 | -.004 | .032 |
|  | social-external HLC | -.004 | .011 | -.045 | -.406 | .685 | -.026 | .017 |
|  | fatalistic-external HLC | -.013 | .008 | -.164 | -1.552 | .124 | -.029 | .004 |
|  | PHQ-9 sum score | .023 | .010 | .224 | 2.169 | .032 | .002 | .043 |
|  | Model 3 Corrected R^2^: .061 | | | | | | | |
| 4 | (Constant) | .015 | .415 |  | .037 | .971 | -.808 | .838 |
|  | Age | -.003 | .004 | -.080 | -.771 | .442 | -.010 | .004 |
|  | Education | .085 | .068 | .124 | 1.242 | .217 | -.051 | .220 |
|  | internal HLC | .014 | .009 | .172 | 1.579 | .118 | -.004 | .032 |
|  | social-external HLC | -.005 | .011 | -.048 | -.450 | .654 | -.026 | .016 |
|  | fatalistic-external HLC | -.013 | .008 | -.164 | -1.554 | .123 | -.029 | .004 |
|  | PHQ-9 sum score | .023 | .010 | .227 | 2.240 | .027 | .003 | .043 |
|  | Model 4 Corrected R^2^: .070 | | | | | | | |
| 5 | (Constant) | -.027 | .402 |  | -.067 | .946 | -.825 | .771 |
|  | Age | -.003 | .004 | -.084 | -.814 | .417 | -.010 | .004 |
|  | Education | .077 | .066 | .113 | 1.171 | .244 | -.053 | .208 |
|  | internal HLC | .013 | .008 | .156 | 1.521 | .131 | -.004 | .030 |
|  | fatalistic-external HLC | -.014 | .008 | -.175 | -1.711 | .090 | -.029 | .002 |
|  | PHQ-9 sum score | .023 | .010 | .229 | 2.280 | .025 | .003 | .043 |
|  | Model 5 Corrected R^2^: .078 | | | | | | | |
| 6 | (Constant) | -.267 | .273 |  | -.979 | .330 | -.809 | .274 |
|  | Education | .082 | .065 | .120 | 1.249 | .215 | -.048 | .212 |
|  | internal HLC | .015 | .008 | .181 | 1.859 | .066 | -.001 | .031 |
|  | fatalistic-external HLC | -.015 | .008 | -.187 | -1.855 | .067 | -.030 | .001 |
|  | PHQ-9 sum score | .026 | .010 | .253 | 2.631 | .010 | .006 | .045 |
|  | Model 6 Corrected R^2^: .081 | | | | | | | |
| 7 | (Constant) | -.072 | .225 |  | -.322 | .748 | -.518 | .373 |
|  | internal HLC | .016 | .008 | .191 | 1.960 | .053 | .000 | .032 |
|  | fatalistic-external HLC | -.016 | .008 | -.209 | -2.109 | .037 | -.032 | -.001 |
|  | PHQ-9 sum score | .025 | .010 | .245 | 2.551 | .012 | .006 | .044 |
|  | Model 7 Corrected R^2^: .076 | | | | | | | |

# Table 3. Coefficients of linear regression with SAMS missing knowledge as dependent variable

| Model | | Unstandardized Coefficients | |  | t | Sig. | 95%CI | |
| --- | --- | --- | --- | --- | --- | --- | --- | --- |
|  |  | B | Std. Error | Std. coefficient B |  |  | Lower Bound | Upper Bound |
| 1 | (Constant) | .274 | .412 |  | .666 | .507 | -.543 | 1.092 |
|  | Gender | -.080 | .088 | -.096 | -.910 | .365 | -.256 | .095 |
|  | Age | .000 | .003 | .005 | .043 | .966 | -.006 | .006 |
|  | Education | -.115 | .055 | -.208 | -2.087 | .039 | -.224 | -.006 |
|  | Number of drugs per day | .029 | .015 | .218 | 1.923 | .057 | -.001 | .060 |
|  | internal HLC | -.002 | .007 | -.029 | -.271 | .787 | -.016 | .012 |
|  | social-external HLC | .007 | .009 | .083 | .750 | .455 | -.011 | .024 |
|  | fatalistic-external HLC | -.015 | .007 | -.241 | -2.275 | .025 | -.028 | -.002 |
|  | HCCQ mean value | .012 | .041 | .030 | .305 | .761 | -.068 | .093 |
|  | PHQ-9 sum score | .015 | .009 | .173 | 1.700 | .092 | -.002 | .032 |
|  | Model 1 Corrected R^2^: .061 | | | | | | | |
| 2 | (Constant) | .284 | .336 |  | .846 | .399 | -.382 | .951 |
|  | Gender | -.080 | .086 | -.095 | -.926 | .357 | -.250 | .091 |
|  | Education | -.115 | .054 | -.209 | -2.110 | .037 | -.223 | -.007 |
|  | Number of drugs per day | .030 | .014 | .220 | 2.125 | .036 | .002 | .057 |
|  | Subsummenscore internal | -.002 | .007 | -.030 | -.281 | .779 | -.016 | .012 |
|  | social-external HLC | .007 | .009 | .083 | .753 | .453 | -.011 | .024 |
|  | fatalistic-external HLC | -.015 | .007 | -.241 | -2.287 | .024 | -.028 | -.002 |
|  | HCCQ mean value | .012 | .040 | .029 | .304 | .762 | -.068 | .092 |
|  | PHQ-9 sum score | .015 | .008 | .172 | 1.746 | .084 | -.002 | .032 |
|  | Model 2 Corrected R^2^:.071 | | | | | | | |
| 3 | (Constant) | .257 | .320 |  | .803 | .424 | -.378 | .891 |
|  | Gender | -.079 | .086 | -.095 | -.923 | .358 | -.249 | .091 |
|  | Education | -.114 | .054 | -.208 | -2.110 | .037 | -.222 | -.007 |
|  | Number of drugs per day | .031 | .013 | .228 | 2.303 | .023 | .004 | .057 |
|  | social-external HLC | .006 | .008 | .072 | .702 | .484 | -.010 | .022 |
|  | fatalistic-external HLC | -.015 | .006 | -.246 | -2.377 | .019 | -.028 | -.003 |
|  | HCCQ mean value | .012 | .040 | .029 | .302 | .763 | -.068 | .092 |
|  | PHQ-9 sum score | .015 | .008 | .173 | 1.763 | .081 | -.002 | .032 |
|  | Model 3 Corrected R^2^: .079 | | | | | | | |
| 4 | (Constant) | .327 | .219 |  | 1.495 | .138 | -.107 | .761 |
|  | Gender | -.083 | .084 | -.099 | -.986 | .326 | -.250 | .084 |
|  | Education | -.115 | .054 | -.208 | -2.128 | .036 | -.221 | -.008 |
|  | Number of drugs per day | .030 | .013 | .225 | 2.294 | .024 | .004 | .057 |
|  | social-external HLC | .006 | .008 | .075 | .740 | .461 | -.010 | .022 |
|  | fatalistic-external HLC | -.015 | .006 | -.241 | -2.369 | .020 | -.028 | -.002 |
|  | PHQ-9 sum score | .015 | .008 | .170 | 1.751 | .083 | -.002 | .031 |
|  | Model 4 Corrected R^2^: .088 | | | | | | | |
| 5 | (Constant) | .415 | .183 |  | 2.269 | .025 | .052 | .778 |
|  | Gender | -.073 | .083 | -.087 | -.878 | .382 | -.237 | .092 |
|  | Education | -.104 | .052 | -.190 | -2.009 | .047 | -.207 | -.001 |
|  | Number of drugs per day | .031 | .013 | .228 | 2.341 | .021 | .005 | .057 |
|  | fatalistic-external HLC | -.014 | .006 | -.217 | -2.255 | .026 | -.025 | -.002 |
|  | PHQ-9 sum score | .015 | .008 | .170 | 1.748 | .083 | -.002 | .031 |
|  | Model 5 Corrected R^2^: .092 | | | | | | | |
| 6 | (Constant) | .399 | .182 |  | 2.193 | .031 | .038 | .759 |
|  | Education | -.109 | .052 | -.198 | -2.106 | .038 | -.211 | -.006 |
|  | Number of drugs per day | .028 | .013 | .205 | 2.186 | .031 | .003 | .053 |
|  | fatalistic-external HLC | -.013 | .006 | -.216 | -2.247 | .027 | -.025 | -.002 |
|  | PHQ-9 sum score | .016 | .008 | .188 | 1.991 | .049 | .000 | .032 |
|  | Model 6 Corrected R^2^: .094 | | | | | | | |

# Table 4. Coefficients of linear regression with SAMS forgetting as dependent variable

| Model | | Unstandardized Coefficients | |  | t | Sig. | 95%CI | |
| --- | --- | --- | --- | --- | --- | --- | --- | --- |
|  |  | B | Std. Error | Std. coefficient B |  |  | Lower Bound | Upper Bound |
| 1 | (Constant) | -.226 | .563 |  | -.402 | .689 | -1.353 | .901 |
|  | Gender | .218 | .131 | .243 | 1.662 | .102 | -.044 | .480 |
|  | Age | -.007 | .004 | -.249 | -1.607 | .113 | -.015 | .002 |
|  | Education | .025 | .076 | .042 | .328 | .744 | -.128 | .178 |
|  | Number of drugs per day | .022 | .021 | .155 | 1.041 | .302 | -.020 | .063 |
|  | internal HLC | .010 | .009 | .148 | 1.006 | .319 | -.009 | .028 |
|  | social-external HLC | .004 | .013 | .042 | .283 | .778 | -.022 | .029 |
|  | fatalistic-external HLC | -.005 | .009 | -.080 | -.530 | .598 | -.023 | .013 |
|  | HCCQ mean value | .064 | .052 | .160 | 1.228 | .224 | -.040 | .168 |
|  | PHQ-9 sum score | .012 | .011 | .153 | 1.142 | .258 | -.009 | .034 |
|  | Model 1 Corrected R^2^: .044 | | | | | | | |
| 2 | (Constant) | -.199 | .551 |  | -.362 | .719 | -1.301 | .903 |
|  | Gender | .231 | .121 | .258 | 1.901 | .062 | -.012 | .474 |
|  | Age | -.007 | .004 | -.252 | -1.636 | .107 | -.015 | .002 |
|  | Education | .029 | .075 | .048 | .383 | .703 | -.121 | .178 |
|  | Number of drugs per day | .022 | .021 | .157 | 1.064 | .292 | -.019 | .063 |
|  | internal HLC | .010 | .009 | .159 | 1.120 | .267 | -.008 | .028 |
|  | fatalistic-external HLC | -.004 | .008 | -.064 | -.460 | .647 | -.021 | .013 |
|  | HCCQ mean value | .066 | .051 | .166 | 1.297 | .200 | -.036 | .168 |
|  | PHQ-9 sum score | .012 | .011 | .152 | 1.140 | .259 | -.009 | .033 |
|  | Model 2 Corrected R^2^: .059 | | | | | | | |
| 3 | (Constant) | -.122 | .508 |  | -.239 | .812 | -1.138 | .895 |
|  | Gender | .237 | .120 | .264 | 1.978 | .053 | -.003 | .476 |
|  | Age | -.007 | .004 | -.261 | -1.727 | .089 | -.015 | .001 |
|  | Number of drugs per day | .022 | .020 | .154 | 1.053 | .297 | -.019 | .062 |
|  | internal HLC | .010 | .009 | .160 | 1.141 | .259 | -.008 | .028 |
|  | fatalistic-external HLC | -.004 | .008 | -.068 | -.495 | .623 | -.021 | .012 |
|  | HCCQ mean value | .067 | .051 | .167 | 1.313 | .194 | -.035 | .168 |
|  | PHQ-9 sum score | .012 | .010 | .145 | 1.106 | .273 | -.009 | .032 |
|  | Model 3 Corrected R^2^: .072 | | | | | | | |
| 4 | (Constant) | -.128 | .505 |  | -.253 | .801 | -1.137 | .882 |
|  | Gender | .248 | .116 | .278 | 2.135 | .037 | .016 | .481 |
|  | Age | -.007 | .004 | -.262 | -1.746 | .086 | -.015 | .001 |
|  | Number of drugs per day | .019 | .020 | .139 | .976 | .333 | -.020 | .059 |
|  | internal HLC | .009 | .008 | .134 | 1.037 | .304 | -.008 | .025 |
|  | HCCQ mean value | .065 | .050 | .163 | 1.289 | .202 | -.036 | .165 |
|  | PHQ-9 sum score | .010 | .010 | .126 | 1.014 | .315 | -.010 | .030 |
|  | Model 4 Corrected R^2^: .084 | | | | | | | |
| 5 | (Constant) | -.091 | .503 |  | -.182 | .857 | -1.098 | .915 |
|  | Gender | .265 | .115 | .296 | 2.304 | .025 | .035 | .495 |
|  | Age | -.005 | .004 | -.202 | -1.476 | .145 | -.013 | .002 |
|  | internal HLC | .007 | .008 | .114 | .889 | .377 | -.009 | .024 |
|  | HCCQ mean value | .060 | .050 | .151 | 1.200 | .235 | -.040 | .160 |
|  | PHQ-9 sum score | .011 | .010 | .141 | 1.135 | .261 | -.009 | .031 |
|  | Model 5 Corrected R^2^: .084 | | | | | | | |
| 6 | (Constant) | .143 | .428 |  | .333 | .740 | -.713 | .999 |
|  | Gender | .272 | .115 | .304 | 2.377 | .021 | .043 | .501 |
|  | Age | -.007 | .003 | -.248 | -1.965 | .054 | -.013 | .000 |
|  | HCCQ mean value | .063 | .050 | .159 | 1.270 | .209 | -.036 | .163 |
|  | PHQ-9 sum score | .011 | .010 | .137 | 1.106 | .273 | -.009 | .031 |
|  | Model 6 Corrected R^2^: .087 | | | | | | | |
| 7 | (Constant) | .319 | .398 |  | .800 | .427 | -.477 | 1.115 |
|  | Gender | .251 | .113 | .281 | 2.220 | .030 | .025 | .477 |
|  | Age | -.008 | .003 | -.283 | -2.306 | .024 | -.014 | -.001 |
|  | HCCQ mean value | .056 | .049 | .140 | 1.126 | .265 | -.043 | .154 |
|  | Model 7 Corrected R^2^: .084 | | | | | | | |
| 8 | (Constant) | .707 | .200 |  | 3.534 | .001 | .307 | 1.106 |
|  | Gender | .216 | .109 | .242 | 1.984 | .052 | -.001 | .434 |
|  | Age | -.008 | .003 | -.299 | -2.451 | .017 | -.015 | -.001 |
|  | Model 8 Corrected R^2^: .080 | | | | | | | |

# Table 5. Elastic net model with SAMS total as dependent variable.

MODEL FIT:

χ²(6) = 455.998, p = 0.008,

Pseudo-R² (Cragg-Uhler) = 0.147,

Pseudo-R² (McFadden) = 0.026,

AIC = 673.421, BIC = 694.952

----------------------------------------------------------------

Est. 2.5% 97.5% t val. p

-------------------- -------- -------- -------- -------- -------

(Intercept) 3.702 -3.510 10.913 1.006 0.317

age -0.036 -0.107 0.035 -0.996 0.321

internal HLC 0.138 -0.029 0.304 1.624 0.108

fatalistic-external HLC-0.133 -0.291 0.026 -1.644 0.103

PHQ9 0.281 0.072 0.491 2.632 0.010

education_low -0.453 -3.152 2.246 -0.329 0.743

education_middle 1.026 -1.276 3.328 0.874 0.384

----------------------------------------------------------------

# Table 6. Elastic net model with SAMS modification as dependent variable.

MODEL FIT:

χ²(2) = 1.802, p = 0.026

Pseudo-R² (Cragg-Uhler) = 0.086

Pseudo-R² (McFadden) = 0.047

AIC = 156.080, BIC = 166.696

------------------------------------------------------------

Est. 2.5% 97.5% t val. p

----------------- -------- -------- ------- -------- -------

(Intercept) 0.414 -0.054 0.883 1.733 0.086

age -0.005 -0.012 0.001 -1.593 0.114

PHQ9 0.017 -0.003 0.037 1.710 0.090

------------------------------------------------------------

# Table 7. Elastic net model with SAMS missing knowledge as dependent variable.

MODEL FIT:

χ²(7) = 2.421, p = 0.030

Pseudo-R² (Cragg-Uhler) = 0.206

Pseudo-R² (McFadden) = 0.137

AIC = 115.811, BIC = 139.950

-----------------------------------------------------------------

Est. 2.5% 97.5% t val. p

--------------------- -------- -------- -------- -------- -------

(Intercept) 0.134 -0.270 0.539 0.652 0.516

Gender_female 0.072 -0.094 0.238 0.853 0.396

Number of drugs 0.031 0.004 0.058 2.236 0.028

internal HLC 0.000 -0.013 0.013 0.018 0.985

fatalistic-external HLC -0.014 -0.026 -0.001 -2.125 0.036

PHQ9 0.015 -0.002 0.031 1.703 0.092

education_low 0.100 -0.109 0.309 0.936 0.351

education_high -0.108 -0.289 0.073 -1.173 0.244

-----------------------------------------------------------------

# Table 8. Elastic net model with SAMS forgetting as dependent variable.

MODEL FIT:

χ²(1) = 0.315, p = 0.190

Pseudo-R² (Cragg-Uhler) = 0.037

Pseudo-R² (McFadden) = 0.023

AIC = 81.698, BIC = 88.356

Standard errors: MLE

---------------------------------------------------------------

Est. 2.5% 97.5% t val. p

-------------------- -------- -------- ------- -------- -------

(Intercept) 0.383 0.212 0.555 4.382 0.000

Gender_female -0.142 -0.356 0.071 -1.310 0.195

---------------------------------------------------------------
